# Rapid reduction of substantially increased myoglobin and creatine kinase levels using a hemoadsorption device (CytoSorb^®^)—A case report

**DOI:** 10.1002/ccr3.5272

**Published:** 2022-01-20

**Authors:** Erich Moresco, Christopher Rugg, Mathias Ströhle, Matthias Thoma

**Affiliations:** ^1^ Department of Anesthesiology and Intensive Care Medicine Medical University of Innsbruck Innsbruck Austria

**Keywords:** acute kidney injury, CytoSorb^®^, myoglobin, renal replacement therapy, rhabdomyolysis

## Abstract

Rhabdomyolysis (RM) is a potentially life‐threatening entity that can lead to acute kidney injury (AKI). Continuous renal replacement therapy (CRRT) alone is known as effective therapy, but the additional use of a hemoadsorber (like CytoSorb^®^) might increase its efficacy.

## Graphical abstract

Early initiation of continuous renal replacement therapy (CRRT) can reduce myoglobin‐induced renal damage in rhabdomyolysis. CRRT or CRRT with the addition of the hemoadsorber CytoSorb^®^ should be considered if available in severe rhabdomyolysis along with adequate fluid replacement to enhance the reduction of myoglobin in plasma.

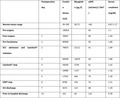

## 1 INTRODUCTION

RM is a clinical entity associated with elevated creatine kinase (CK), myoglobin and electrolyte misbalance caused by major trauma, muscle injury, or other factors.^1^ Besides preventing further muscle injury, treatment primarily targets a reduction of these potentially nephrotoxic agents (CK, myoglobin) and a correction of the accompanying metabolic acidosis and electrolyte disorders. If standard therapy with intravenous fluids, osmodiuretics, and bicarbonate fails, CRRT may liberally be indicated in order to prevent further kidney injury, although mortality rates remain unchanged.^2^ CytoSorb^®^ (Cytosorbents Corporation, Monmouth Junction; New Jersey, USA) is an adsorption device which can be used as standalone therapy or in combination with CRRT.^3^ It was approved in the European Union in 2011, primarily for the reduction of mid‐molecular weight inflammatory mediators.^4^ Since then, its application range has grown from septic patients, where a rapid hemodynamic stabilization and an increased survival were observed^5^ to drug removal and cardiac surgery patients.^3^ Until now, only a few papers have reported on the promising benefits of the use of CytoSorb^®^ in RM.^6^, ^7^ In this case report, we describe the successful use of CytoSorb^®^ in rapidly reducing CK and myoglobin levels in a patient suffering RM following sport trauma and successive surgeries.

## 2 CASE REPORT

We describe a case of severe RM in a 24‐year‐old, healthy male patient who had compartment syndrome. He suffered a knee dislocation due to a sport trauma and received knee surgery in combined general anesthesia with peripheral nerve block (preoperative blood analysis was as follows: CK levels 1300 U/l, estimated glomerular filtration rate (eGFR) 82 ml/min/1.73m², serum creatinine (SC) 1.1 mg/dl; see Table 1). The procedure was performed in a peripheral clinic, lasted 5 h, and included the use of a tourniquet on the thigh for approximately 2 hours. The following day after surgery, the patient developed a swollen thigh with brown‐colored urine. Laboratory results showed elevated levels of CK (89,968 U/l) and myoglobin (>500 µg/l, unspecified) with an already impaired kidney function (eGFR 50 ml/min/1.73m², SC 1.68 mg/dl; see Table 1). The patient was promptly transferred to our hospital for fasciotomy and hematoma evacuation. Postoperatively, the patient was admitted to our ICU for further care.

**TABLE 1:** Levels of creatine kinase, myoglobin, creatinine, and eGFR during hospitalization

	Postoperative day	Creatine kinase [U/l]	Myoglobin [µg /l]	eGFR [ml/min/1.73m²]	Serum creatinine [mg/dl]
Normal values range		39–190	28–72	<60	0.67–1.17
Pre‐surgery		1300,8		82	1.1
Post‐surgery	1	70647		60	1.44
Pre‐Fasciotomy	1	89968		50	1.68
ICU admission and CytoSorb^®^ initiation	2	79833	22111	41	1.99
	2	60938	14679	44	1.88
CytoSorb^®^ stop	3	34630	3730	61	1.42
	4	24089	1147	76	1.18
	5	17541	465	74	1.20
CRRT stop	6	8700	226	74	1.21
ICU discharge	7	3673	123	68	1.29
Prior to hospital discharge	15	161	28	122	0.78

ICU admission myoglobin and CK levels were 15,993 µg/l and 76,182 U/l, respectively (see Table 1).

Due to the degree of CK elevation, continuous veno‐venous hemofiltration (cvvHF; AN‐69 ST membrane, Gambro Prismaflex^®^ Baxter, Deerfield, USA) carrying a CytoSorb^®^ adsorber column, was promptly initiated on ICU admission (blood flow: 150 ml/min, resulting pre‐dilution fluid: 1500 ml/h, post‐dilution fluid: 1000 ml/h, withdrawal: 100 ml/h). Trisodium citrate was used as regional anticoagulation. No other indications than carrying CytoSorb^®^ necessitated a start of CRRT.

Within 24 hours, a reduction in CK levels by more than 50% and myoglobin levels by more than 80% was achieved (see Table 1). CytoSorb^®^ therapy included one column for 24 h. Thereafter, CRRT therapy alone was continued up to the 6th postoperative day.

In addition, osmodiuretics (mannitol 15%) were administered intravenously on the first 2 days on ICU (total amount: 500 ml). Additional bicarbonate was not given. Resuscitation fluid therapy was conducted with colloids (Gelofusine^®^ 4%, B. Braun, Melsungen, Germany), maintenance fluids.

included balanced crystalloids (ELO‐MEL isotone Fresenius Kabi, Bad Homburg, Germany). The patient was scheduled to leave the intensive care unit on the 6^th^ postoperative day. He was eventually transferred to the intermediate care on the 7th and discharged from hospital on the 16th postoperative day. The day before hospital discharge laboratory results had shown a fully recovered renal function (eGFR 122 ml/min/m², SC 0.78 mg/dl) and normal levels of creatine kinase (161 U/l) and myoglobin (28 µg/l).

## 3 DISCUSSION

Rhabdomyolysis is characterized by muscle cell death and therefore elevated CK and myoglobin levels in the blood which in some cases can lead to AKI.^8^ If available and possible, surgical intervention to reduce further muscle damage should be performed (ie fasciotomy). If therapy with osmodiuretics and aggressive fluid therapy fails, CRRT is a further therapeutic option when available.^2^ In our case, the immediate use of CRRT with the addition of the hemoadsorber CytoSorb^®^ led to a highly efficient reduction of CK and myoglobin concentrations in the blood (Table 1). Notable is the initial decrease of myoglobin which was faster than CK. This is in accordance with Dilken et al., who described a significant reduction when using CytoSorb^®^.^7^ As myoglobin is a relatively small molecule with a molecular mass of 17 kilodalton (kDa), it is presumed to be effectively adsorbed by CytoSorb^®^. Daum et al. determine that CK is unlikely to be adsorbed by CytoSorb^®^ due to its relatively large mass (82 kDa).^9^ Due to a partly known adsorption effect of the AN‐69 ST membrane and its sieving.

coefficient of 0.58 a constant amount of myoglobin can be removed from the plasma. When.

myoglobin in the plasma is excessively increased as it was in this case the additional use of a commercially available adsorption system might help to reduce plasma peak levels of myoglobin. This could lead to less nephrotoxicity and could faster improve the patient's condition. From this point of view, it might justify to implement CRRT as well as a hemoadsorber like CytoSorb^®^ in the future as soon as excessive myoglobin peak levels are reached and the cause for them is known. The early use of CRRT.

alone or CRRT with the addition of the hemoadsorber CytoSorb^®^ could help to prevent AKI instead of just treating the AKI afterward. Nevertheless, the treating intensivist has to be aware that additional costs when using CRRT and Cytosorb^®^ will arise. This has to be waged against the risk of persisting renal failure, especially in young as this will exceed the primary cost multiple times.

There are some major limitations regarding this case, the use of Cytosorb seemed us justified as there was a considerable risk of persisting AKI in a young sportive patient.

It is noteworthy to mention that the patient developed pronounced RM after a 5‐hour surgery. The transfer to our unit was delayed, despite already elevated levels of CK and myoglobin and a brown‐colored urine. Due to the peripheral nerve catheter and continuous analgesia, pain was not reported.

We presume that RM was caused by compartment syndrome due to the length of surgery or the prolonged use of operative tourniquet. RM following surgery is a quite uncommon event, but nonetheless increased intracompartmental pressure and muscular ischemia can lead to RM.^10^, ^11^ Prolonged duration of surgery and intraoperative unusual positions are described as risk factors.^12^ Both surgeon and anesthesiologist should be aware of the risk, monitor the patient carefully, and initiate aggressive therapy if the diagnose is confirmed. The patient's kidney function did recover.

Risks for non‐recovery are known to be high age, chronic kidney disease, higher severity of AKI, delayed nephrology consultation, and therefore delayed onset of therapy.^13^, ^14^

## CONFLICT OF INTERESTS

None declared.

## AUTHORS CONTRIBUTIONS

EM, CR, MS, and MT contributed to conception, design, and analysis. EM and MT contributed to data acquisition, and drafted the manuscript. All authors critically revised the manuscript, contributed to interpretation, gave final approval, and agreed to be accountable for all aspects of the work ensuring integrity and accuracy.

## ETHICS APPROVAL

Not applicable.

## CONSENT

Written informed consent was obtained from the patient for publication of this case report. A copy of the written consent is available for review by the Editor in Chief of this journal upon request.

## Data Availability

Data are available on reasonable request.
